# Integrated microbiome and metabolome analysis reveals the potential therapeutic mechanism of *Qing-Fei-Pai-Du* decoction in mice with coronavirus-induced pneumonia

**DOI:** 10.3389/fcimb.2022.950983

**Published:** 2022-08-26

**Authors:** Gaosong Wu, Wendan Zhang, Ningning Zheng, Xianpeng Zu, Saisai Tian, Jing Zhong, Yuhao Zhang, Jingyu Liao, Lili Sheng, Guanbo Ge, Houkai Li, Weidong Zhang

**Affiliations:** ^1^ Institute of Interdisciplinary Integrative Medicine Research, Shanghai University of Traditional Chinese Medicine, Shanghai, China; ^2^ School of Pharmacy, Shanghai University of Traditional Chinese Medicine, Shanghai, China; ^3^ School of Pharmacy, Second Military Medical University, Shanghai, China

**Keywords:** *Qing-Fei-Pai-Du* decoction, coronavirus disease 2019, gut microbiota, metabolome, immune, inflammation

## Abstract

Current studies have shown that gut microbiota may be closely related to the severity of coronavirus disease 2019 (COVID-19) by regulating the host immune response. *Qing-Fei-Pai-Du* decoction (QFPDD) is the recommended drug for clinical treatment of patients with COVID-19 in China, but whether it exerts a therapeutic effect by modulating the immune response through gut microbiota remains unclear. In this study, we evaluated the therapeutic effects of QFPDD in pneumonia model mice and performed 16S rRNA sequencing and serum and lung tissue metabolomic analysis to explore the underlying mechanisms during the treatment. Then, Spearman correlation analysis was performed on gut microbiome, serum metabolome, and immune-inflammation-related indicators. Our results suggest that QFPDD can restore the richness and diversity of gut microbiota, and multiple gut microbiota (including *Alistipes*, *Odoribacter*, *Staphylococcus*, *Lachnospiraceae_NK4A136_group Enterorhabdus*, and *unclassified_f_Lachnospiraceae*) are significantly associated with immune-inflammation-related indicators. In addition, various types of lipid metabolism changes were observed in serum and lung tissue metabolome, especially glycerophospholipids and fatty acids. A total of 27 differential metabolites (DMs) were significantly correlated with immune-inflammation-related indicators, including 9 glycerophospholipids, 7 fatty acids, 3 linoleic acid, 2 eicosanoids, 2 amino acids, 2 bile acids, and 2 others. Interestingly, these DMs showed a good correlation with the gut microbiota affected by QFPDD. The above results suggest that QFPDD can improve the immune function and reduce inflammation in pneumonia model mice by remodeling gut microbiota and host metabolism.

## 1 Introduction

Coronavirus disease 2019 (COVID-19) remains a global pandemic, with more than 517 million confirmed cases and 6.3 million deaths (as of May 2022) (WHO, 2022). COVID-19, also known as severe acute respiratory syndrome coronavirus 2 (SARS-CoV-2), is a novel coronavirus serotype (Zhou et al., 2020). Due to the lack of immunity to SARS-CoV-2 in the population and its high transmissivity, the virus rapidly spread worldwide and was declared as a global pandemic by the World Health Organization (WHO) in March 2020 (Petersen et al., 2020; WHO, 2022). Recent studies revealed the lipid metabolism (including glycerophospholipids, steroid hormones, and sphingolipids) disordered in COVID-19 patients is closely associated with the immune system (Shen et al., 2020; Song et al., 2020), and the gut microbiome may be associated with COVID-19 severity by modulating host immune responses (Zuo et al., 2020; Yeoh et al., 2021). From the perspective of traditional Chinese medicine (TCM), COVID-19 belongs to the category of TCM epidemic, which is manifested as the pathological characteristics of dampness toxin in TCM syndromes (Luo et al., 2020).

TCM has rich clinical experience and effective formulas on the prevention and treatment of epidemic diseases. In China, *Qing-Fei-Pai-Du* decoction (QFPDD) is the common formula for the treatment of COVID-19 in different stages and has been proven safe and effective in clinical trials in multi-provincial (Ren et al., 2020; Shi et al., 2020; Wang et al., 2021), which is recommended for use in patients in the seventh edition of the COVID-19 guideline. As a super combination of 20 herbs and a mineral drug (gypsum fibrosum), more than 450 prototype components and metabolic active components have been identified in QFPDD (Liu et al., 2021). By means of computer-aided drug design, network pharmacology, and molecular docking, the physiological processes involved in the pharmacodynamic mechanism of QFPDD include immune, inflammation regulation, neuroprotection, and reduction of lung injury in the treatment of COVID-19 patients (Ren et al., 2020). However, due to the lack of experimental verification in these studies, the credibility of these results is reduced. Recently, we demonstrated that QFPDD can improve the immune function and reduce liver damage and inflammation by hepatic single-cell RNA sequencing (Tian et al., 2022). However, it is unclear whether the regulation of the immune system by QFPDD is related to the gut microbiota.

In order to explain these important issues, we performed correlation analysis of immune-inflammation-related indicators, gut microbiome, and metabolome data to further reveal the mechanism of QFPDD in the treatment of COVID-19. We first evaluated the effect of QFPDD on the histopathology of mice with pneumonia and then 16S rRNA gene sequencing and Liquid chromatography-tandem mass spectrometry (LC-MS) to reveal changes in the gut microbiota and metabolome. Finally, an integrated analysis of the gut microbiome and serum metabolome with immune-inflammatory-related indicators was performed to reveal the potential therapeutic effects of QFPDD. Our results provide new evidence about how QFPDD improved COVID-19 patients by remodeling the gut microbiota and affecting metabolism, emphasizing the important role of the gut microbiota in the treatment of COVID-19 patients.

## 2 Materials and methods

### 2.1 Preparation and analysis of *Qing-Fei-Pai-Du* decoction

The lyophilized powder of QFPDD used in the experiment was provided by JOINTOWN Pharmaceutical Group (Wuhan, China; batch number: JZT-QFPDT-0318-PG-0321). Detailed chemical constituents can be found in our previous publication (Liu et al., 2021).

### 2.2 Animal and treatment

Male BALB/c mice (10–12 g) were purchased from Beijing HFK Bioscience Co., Ltd. Human coronavirus 229E (HCoV-229E) was provided by the Institute of Medicinal Biotechnology Chinese Academy of Medical Sciences. Real-time PCR kit (lot: p20191201) for HCoV-229E was purchased from Liferiver Biotechnology Corporation (Shanghai). The animal experiments were conducted in the ABSL-2 laboratory of the Academy of Chinese Medical Sciences under the guidance of Prof. Xiaolan Cui. The study was approved and monitored by the Animal Care and Use Committee of the Institute of Chinese Materia Medica, China Academy of Chinese Medical Sciences.

After 1 week of adaptive feeding, 50 mice were randomly divided into five groups, 10 mice/group. The groups were named as control group (Con), cold-humid environment combined with virus infection (Mod) group, low QFPDD group (QFPDD_L, with a dosage of 0.88 g/kg), middle QFPDD group (QFPDD_M, with a dosage of 1.76 g/kg), and high QFPDD group (QFPDD_H, with a dosage of 3.52 g/kg). Mice except for the Con group were placed daily in an artificial climate chamber with relative humidity of 90% ± 3% and temperature of 4°C ± 2°C for 4 h for 7 days. Mice except for the Con group were anesthetized with ether, and 50 μl of HcoV-229E (100TCID50) was used to infect mice by nose-dripping method on the fifth and sixth day; mice in the Con group were infected with culture solution in parallel. On the fourth day of infection, blood was collected from all mice through the orbit for metabolome analysis, lung tissue was collected for histopathological and metabolomic analysis, and cecal contents were collected for gut microbiota sequencing.

### 2.3 Histological analysis of lung tissue with H&E staining

Lung tissues were fixed with 10% neutral formalin for 48 h, embedded in paraffin. A microtome was performed to slice the formed paraffin blocks into 4–5-μm sections at multiple levels, and then the sections were stained with hematoxylin–eosin (H&E) staining.

### 2.4 Serum and lung tissue sample preparation and metabolome analysis

Samples were prepared according to the method of the **Supplementary Materials**; sample analysis was performed on a Shimadzu HPLC system equipment with SCIEX Triple TOF 5600^+^. Detailed sample processing, instrument parameter settings, and data acquisition and analysis can be found in the **Supplementary Materials**.

### 2.5 16S rRNA sequencing

Microbial community genomic DNA was extracted from 15 samples using EZNA ^®^ soil DNA Kit (Omega Bio-tek, Norcross, GA, USA) according to the manufacturer’s instructions. Qualified DNA samples were applied for amplification of 16S rDNA V3-V4 region using the universal primers 338F and 806R. More detailed PCR, sequencing, and data analysis are described in the **Supplementary Materials**.

### 2.6 Statistical analysis

Data are shown as means ± SEM. Multiple comparisons of other data were performed by using one-way ANOVA. Comparisons between two groups were performed using Student’s *t*-test; *p* < 0.05 was considered statistically significant.

## 3 Results

### 3.1 *Qing-Fei-Pai-Du* decoction improves the pathological symptoms of pneumonia model mice

**Figure 1:**
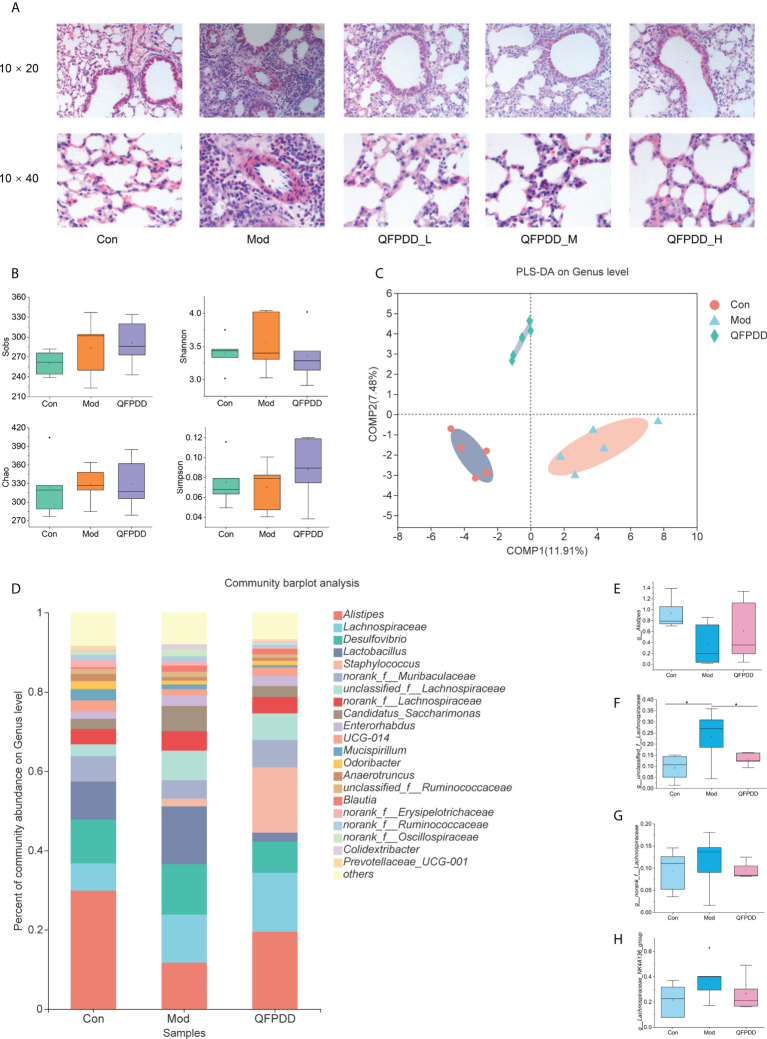
Pathological alterations, gut microbiome diversity, and structural analysis. **(A)** Effect of different doses of *Qing-Fei-Pai-Du* decoction (QFPDD) on lung histopathology. **(B)** Species diversity differences between the Con, Mod, and QFPDD groups were estimated by the observed species, Sobs, Chao, Shannon, Simpson. **(C)** Supervised partial least squares discriminant analysis (PLS-DA) showing the discrimination between the sample groups. **(D)** Bacterial taxonomic profiling in the genus level of gut microbiota in the Con, Mod, and QFPDD groups. **(E–H)** Box plots of relative abundance changes of *Alistipes*, *unclassified_f_Lachnospiraceae*, *norank_f_Lachnospiraceae*, and *Lachnospiraceae_NK4A136_group* in Con, Mod, and QFPDD, *p < 0.05.

To evaluate the effect of QFPDD on pneumonia mice, we firstly analyzed the HcoV-229E nucleic acid expression level in mice and found comparable results among Mod, QFPDD_L, QFPDD_M, and QFPDD_H (**Figure S1**). The histological analysis showed that some extent of lung tissue damage was present in the Mod group, which was characterized by the large number of proliferative cells and lymphocyte exudation, large area of mucus and red blood cell exudation, vascular engorgement, and inflammation around the bronchiole, suggesting that a cold-humid environment combined with virus infection led to histological damage in the lung tissue. After treatment with QFPDD_L, QFPDD_M, and QFPDD_H, the pathological symptoms of lung tissue were alleviated to varying degrees (**Figure 1A**). To study further the effects of QFPDD on the gut microbiota and metabolic profiles, we selected the Con, Mod, and QFPDD_L groups for gut microbiome and metabolome analysis.

### 3.2 *Qing-Fei-Pai-Du* decoction alters bacterial diversity of the gut microbiome

A total number of 2,150,648 high-quality 16S rRNA reads were obtained from the microbiome data, the median reading of each sample was 53,766.2 (range 43,237–60,542), and 472 operational taxonomic units (OTUs) were obtained (**Table S1**). The species accumulation curve (**Figure S2A**) and the rarefaction curve (**Figure S2B**) of all samples supported the adequacy of the sampling efforts. Furthermore, the rank abundance distribution curves (**Figure S2C**) indicated that the richness and relative bacterial imbalance were increased in the Mod group compared with those in the Con group.

In order to assess the differences in bacterial diversity, we compared the α and β diversity among the three groups. Compared with the Con group, Mod and QFPDD have no significant differences in multiple α indexes (Chao, Shannon, Sobs, and Simpson) (**Figure 1B**). Both principal component analysis (PCA) and principal coordinate analysis (PCoA) plots revealed a separation between the three groups (**Figures S3A, S3B**), especially the partial least squares-discriminant analysis (PLS-DA) plots revealed a clear separation between the three groups (**Figure 1C**). These results suggest that the diversity of the gut microbiota could be influenced by QFPDD.

### 3.3 *Qing-Fei-Pai-Du* decoction alters the composition of the gut microbiome

The relative proportions of the three groups of dominant taxa at the phylum level were assessed by microbial taxon assignment. We observed that there were considerable differences in gut microbiota in each group of samples (**Figure S3C**). Ten phyla were identified in each group. Firmicutes was the most predominant phylum, accounting for 39.9%, 57.72%, and 56.29% of the Con, Mod, and QFPDD groups, respectively. In addition, Bacteroidota (18.34% vs. 40.53%) significantly decreased in the Mod group compared with the Con group, while QFPDD could significantly enrich Bacteroidota (28.85% vs. 18.34%) compared with the Mod group.

In order to intuitively compare the differences of microbial communities among the three groups, Welch’s t-test was performed for different taxonomic levels. At the phylum level, the abundance of Firmicutes (*p* < 0.001) in the Mod group was significantly higher than that in the Con group (**Figure S3D**), while there was no significant difference between the Mod group and the QFPDD group. At the genus level, a total of 111 genera were found in the three groups. Of these discriminatory taxa, there were 21 genera with abundance over 1% (**Figure 1D**), *Alistipes* was found to be less abundant in the Mod group than that in the Con group, while *unclassified_f_Lachnospiraceae*, *Lachnospiraceae_NK4A136_group*, and *norank_f_Lachnospiraceae* were found to be more abundant in the Mod group than those in the Con group. The above bacterial levels could be restored to varying degrees after QFPDD treatment (**Figures 1E–H**).

**Figure 2:**
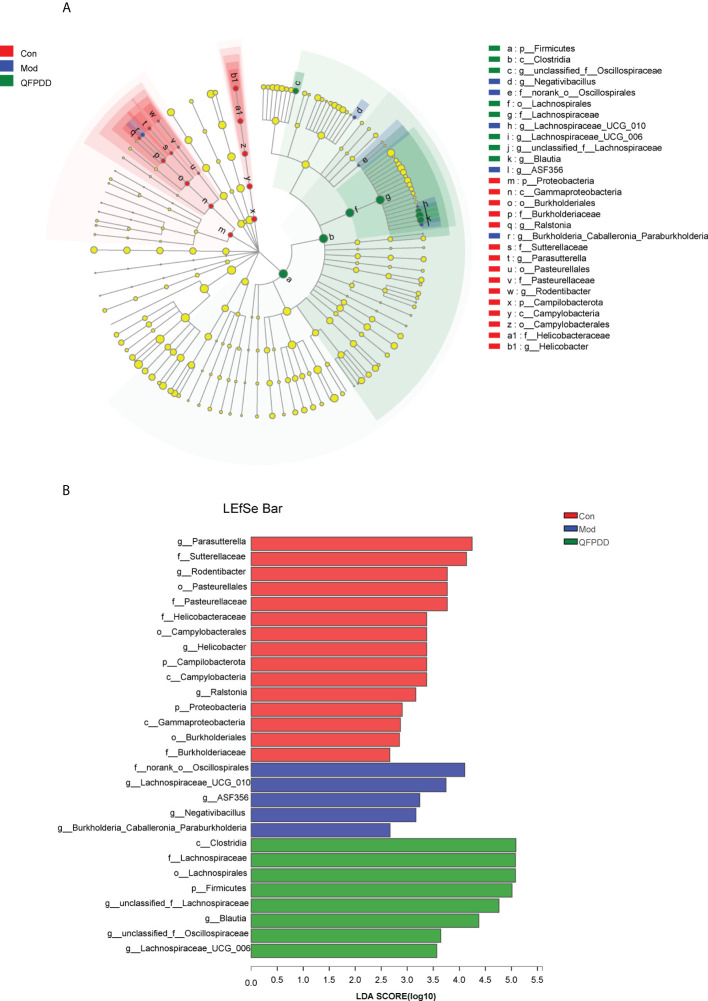
Linear discriminant analysis (LDA) integrated with effect size (LEFSe). **(A)** Cladogram indicating the phylogenetic distribution of microbiota correlated with the Con, Mod, and QFPDD groups. **(B)** The differences in abundance between the Con, Mod, and QFPDD groups.

To identify specific bacteria associated with QFPDD, we performed linear discriminant analysis (LDA) integrated with effect size (LEfSe) to identify the predominant taxon. We identified 28 discriminatory OTUs as key discriminants, Firmicutes (*Lachnospirales*) was significantly overrepresented (all LDA scores >5) in the QFPDD group, whereas Campilobacterota and Proteobacteria (*Gammaproteobacteria*) were the most abundant microbiota in the Con group (LDA scrores >3.6) (**Figure 2A**). Further through LDA, 15 OTUs were enriched in the Con group, 5 OTUs were enriched in the Mod group, and 8 OTUs were enriched in the QFPDD group (**Figure 2B**). These results indicated that QFPDD intervention could improve the gut microbiome richness in mice.

### 3.4 Effects of *Qing-Fei-Pai-Du* Decoction Treatment on Serum and Lung Tissue Metabolome

Gut microbiota plays an important role in host metabolism. To evaluate the metabolic changes caused by gut microbiota after QFPDD treatment, we performed metabolome analysis of serum and lung tissue by LC-MS platform.

#### 3.4.1 *Qing-Fei-Pai-Du* decoction altered the lung metabolome

**Figure 3:**
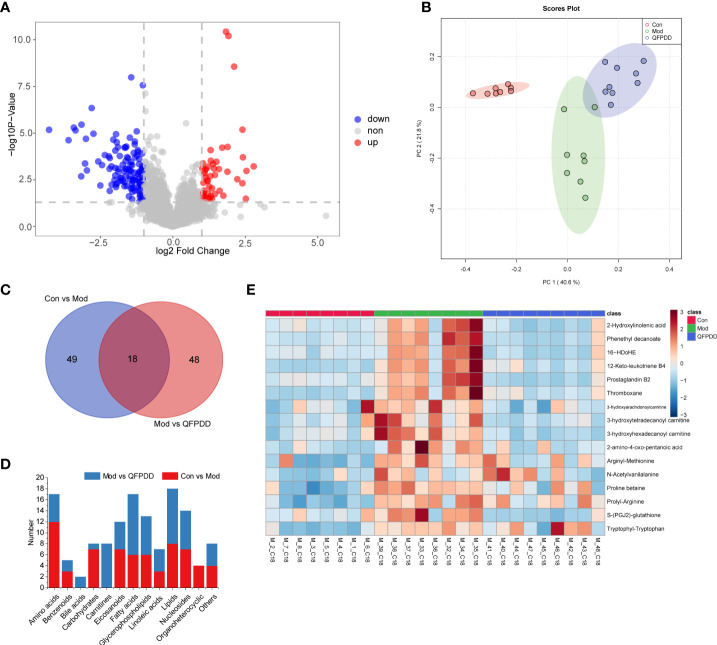
Metabolome analysis of lung tissue. **(A)** Volcano plot showing changes in differential metabolites (DMs) in the Mod and QFPDD groups. **(B)** The principal component analysis (PCA) plots established based on DMs in the Con, Mod, and QFPDD groups. **(C)** Venn diagram of DMs in the Con, Mod, and QFPDD groups. **(D)** The categories and number of DMs obtained from the Con, Mod, and QFPDD groups. **(E)** Heatmap diagram of DMs in the Con, Mod, and QFPDD groups.

In the lung tissue metabolome, a total of 327 dysregulated metabolites were found in the Mod group [Student’s *t*-test, *p* value <0.05, fold change (FC) >2, Mod vs. Con], of which 210 metabolites were upregulated and 117 metabolites were downregulated (**Figure S4A**). A total of 67 differential metabolites (DMs) (*p* < 0.05, FC >2, and variable importance in projection (VIP) >1.5) were identified by Progenesis QI (Waters, Milford, MA, USA) based on the Human Metabolome Database (HMDB) (https://hmdb.ca/), Kyoto Encyclopedia of Genes and Genomes (KEGG) (https://www.kegg.jp/), Lipid metabolites and pathways strategy (LIPIDMAPS) (https://www.lipidmaps.org/), and METLIN (https://metlin.scripps.edu) databases. The volcano diagram showed that QFPDD significantly changed the metabolic profile of the Mod group after intervention. Compared with the Mod group, a total of 171 dysregulated metabolites were found in the Mod group (Student’s *t*-test, *p* values <0.05, FC >2, QFPDD vs. QFPDD), of which 52 metabolites were upregulated and 119 metabolites were downregulated (**Figure 3A**). To maximize identification of DMs in QFPDD, we constructed a PCA model. The lung metabolome of QFPDD was clearly separated from the Mod group on the PCA score plot (**Figure 3B**); based on the DMs obtained by Con, Mod and QFPDD can make a clear separation among the three groups. Of the 171 metabolites, 66 DMs were with VIP >1.5, and the structures were confirmed by Progenesis QI; the independent and shared DMs between them were shown in **Figure 3C**. As shown in **Figure 3D**, QFPDD could significantly affect various types of metabolites, especially fatty acids, carnitines, nucleosides, and glycerophospholipids. The 16 metabolites were significantly affected (false discovery rate (FDR) <0.05, FC >2), including 2 linoleic acids (2-hydroxylinoleic acid and phenethyl decanoate), 1 fatty acid (16-HDOHE), 3 eicosanoids (12-keto-leukotriene B4, prostaglandin B2, and thromboxane), 3 carnitines (3-hydroxyarachidonoylcarnitine, 3-hydroxytetradecanoyl carnitine, and 3-hydroxyhexadecanoyl carnitine), and 7 amino acids [2-amino-4-oxo-pentanoic acid, arginyl-methionine, N-acetylvanilalanine, proline betaine, prolyl-arginine, S-(PGJ2)-glutathione, and tryptophyl-tryptophan] (**Figure 3E**).

#### 3.4.2 *Qing-Fei-Pai-Du* decoction altered the serum metabolome

**Figure 4:**
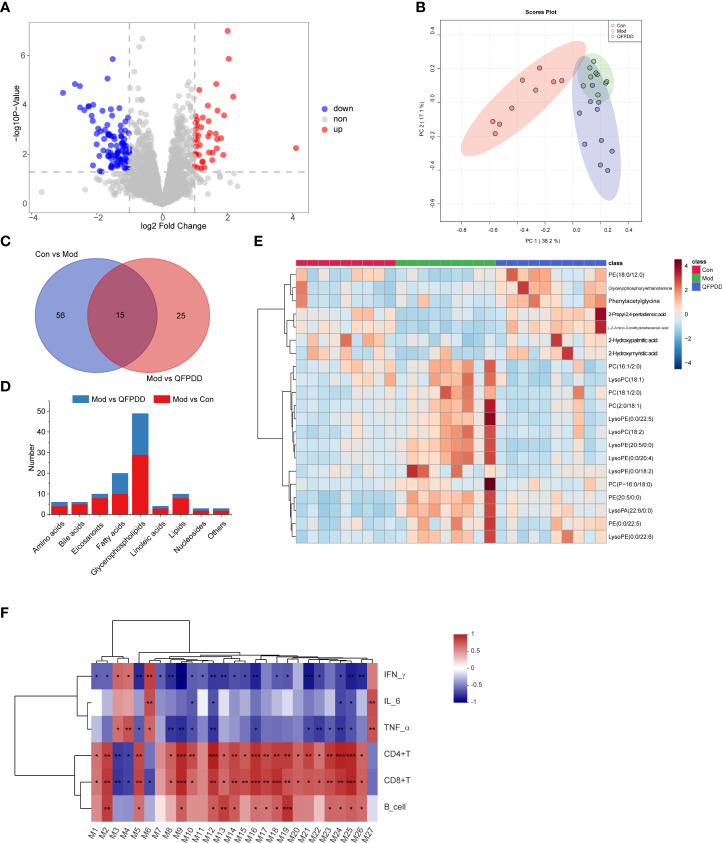
Metabolome analysis of the serum. **(A)** Volcano plot showing changes in DMs in the Mod and QFPDD groups. **(B)** The principal component analysis (PCA) plots established based on DMs in the Con, Mod, and QFPDD groups. **(C)** Venn diagram of DMs in the Con, Mod, and QFPDD groups. **(D)** The categories and number of DMs obtained from the Con, Mod, and QFPDD groups. **(E)** Heatmap diagram of DMs in the Con, Mod, and QFPDD groups. **(F)** Serum differential metabolites significantly correlated with immune and inflammatory indicators. Significance levels are indicated as follows: *, p < 0.05; **, p < 0.01, ***, p < 0.001.

In the serum metabolome, a total of 200 dysregulated metabolites were found in the Mod group (Student’s *t*-test, *p* values <0.05, FC >2, Mod vs. Con), of which 82 metabolites were upregulated and 118 metabolites were downregulated (**Figure S4B**). A total of 71 DMs (*p* < 0.05, FC >2, VIP >1.5) were identified by Progenesis QI. The volcano diagram showed that QFPDD significantly changed the metabolic profile of the Mod group after intervention. Compared with the Mod group, a total of 138 dysregulated metabolites were found in the Mod group (Student’s *t*-test, *p* values <0.05, FC >2, QFPDD vs. QFPDD), of which 43 metabolites were upregulated and 95 metabolites were downregulated (**Figure 4A**). To maximize identification of DMs in QFPDD, we constructed a PCA model. The serum metabolome of QFPDD was clearly separated from the Mod group on the PCA score plot (**Figure 4B**); based on the DMs obtained by Con, Mod and QFPDD can make a clear separation among the three groups. Of the 138 metabolites, 40 DMs were with VIP >1.5, and the structures were confirmed by Progenesis QI; the independent and shared DMs between them were shown in **Figure 4C**. As shown in **Figure 4D**, QFPDD could significantly affect various types of metabolites, especially glycerophospholipids and fatty acids. The 21 metabolites were significantly affected (FDR <0.05, FC >2), including 16 glycerophospholipids [lysoPC(18:2), lysoPE(20:5/0:0), lysoPE(0:0/20:4), PC(16:1/2:0), glycerylphosphorylethanolamine, PC(P-16:0/18:0), PE(0:0/22:5), lysoPE(0:0/22:5), lysoPE(0:0/18:2), PE(20:5/0:0), lysoPA(22:6/0:0), PC(18:1/2:0), lysoPC(18:1), PC(2:0/18:1), lysoPE(0:0/22:6), and PE(18:0/12:0)], 3 fatty acids (2-hydroxypalmitic acid, 2-hydroxymyristic acid, and 2-propyl-2,4-pentadienoic acid), and 2 amino acids (phenylacetylglycine and L-2-amino-3-methylenehexanoic acid) (**Figure 4E**).

#### 3.4.3 The effect of *Qing-Fei-Pai-Du* decoction is associated with lipid metabolism-related metabolic pathway

Our previous studies have shown that QFPDD can improve the immune function and reduce inflammation (Tian et al., 2022). In order to determine which metabolites are associated with the effects of QFPDD on improving immunity and inflammation, we performed a correlation analysis on serum DMs and immune-inflammation-related indicators. As shown in **Figure 4F**, a total of 27 DMs were significantly correlated with immune-inflammation-related indicators, including 9 glycerophospholipids [glycerophosphocholine, LysoPC(15:0), LysoPC(O-18:0), PA(O-16:0/12:0), PA(P-16:0/14:1), PC(20:0/0:0), PE(17:1/19:0), PS(20:0/0:0), and PE(O-20:0/18:3)], 7 fatty acids (12-hydroxyicosanoic acid, 2-hydroxypalmitic acid, 6-hydroxyoctadecanoic acid, 9,10-DHOME, 9-heptadecenoic acid, eicosadienoic acid, and octadecanedioic acid), 3 linoleic acids (13-HOTE, 4,8,12,15-octadecatetraenoic acid, and alpha-linolenic acid), 2 eicosanoids [prostaglandin F2beta and 12(S)-leukotriene B4], 2 amino acids (isoleucyl-hydroxyproline and N-acetyl-L-histidine), 2 bile acids (chenodeoxycholic acid and taurochenodesoxycholic acid), and 2 others (inosine and 13-cis-retinoic acid) (**Table 1**). The above results showed that the effect of QFPDD on immune and inflammatory response in mice was closely related to lipid metabolism, especially glycerophospholipids and fatty acids.

**Table 1:** Differential metabolites significantly associated with immune-inflammatory indicators in the serum metabolome.

No.	Metabolites	Class	HMDB ID	Adducts	Formula	Mass Error (ppm)
**M1**	Isoleucyl-Hydroxyproline	Amino acids	HMDB0028908	M+K	C11H20N2O4	9.6
**M2**	N-Acetyl-L-histidine	Amino acids	HMDB0032055	M+FA-H	C8H11N3O3	8.6
**M3**	Chenodeoxycholic acid	Bile acids	HMDB0000518	M+NH4	C24H40O4	0.6
**M4**	Taurochenodesoxycholic acid	Bile acids	HMDB0000951	M+H, M+Na	C26H45NO6S	-1.2
**M5**	Prostaglandin F2beta	Eicosanoids	HMDB0001483	M-H	C20H34O5	-1.6
**M6**	12-hydroxyicosanoic acid	Fatty acids	HMDB0061664	M+NH4	C20H40O3	-0.2
**M7**	2-Hydroxypalmitic Acid	Fatty acids	HMDB0031057	M-H	C16H32O3	1.6
**M8**	6-Hydroxyoctadecanoic acid	Fatty acids	HMDB0112193	M-H	C18H36O3	-0.7
**M9**	9,10-DHOME	Fatty acids	HMDB0004704	M+Na	C18H34O4	6.8
**M10**	9-Heptadecenoic acid	Fatty acids	HMDB0031046	M+FA-H	C17H32O2	-0.6
**M11**	Eicosadienoic acid	Fatty acids	HMDB0005060	M+Na	C20H36O2	9.7
**M12**	Octadecanedioic acid	Fatty acids	HMDB0000782	M+H	C18H34O4	0.3
**M13**	Glycerophosphocholine	Glycerophospholipids	HMDB0000086	M+H	C8H20NO6P	0.1
**M14**	LysoPC (15:0)	Glycerophospholipids	HMDB0010381	M+H	C23H48NO7P	0.3
**M15**	LysoPC (O-18:0)	Glycerophospholipids	HMDB0011149	M+Na	C26H56NO6P	-0.4
**M16**	PA (O-16:0/12:0)	Glycerophospholipids	NA	M-H	C31H63O7P	-0.7
**M17**	PA (P-16:0/14:1)	Glycerophospholipids	NA	M-H	C33H63O7P	-0.8
**M18**	PC (20:0/0:0)	Glycerophospholipids	NA	M+H	C28H58NO7P	0.7
**M19**	PE (17:1/19:0)	Glycerophospholipids	NA	M+FA-H	C41H80NO8P	0.6
**M20**	PS (20:0/0:0)	Glycerophospholipids	NA	M+H	C26H52NO9P	-2.2
**M21**	PE (O-20:0/18:3)	Glycerophospholipids	NA	M+FA-H	C43H82NO7P	-0.7
**M22**	13-HOTE	Linoleic acids	HMDB0010203	M+H	C18H30O3	1.1
**M23**	4,8,12,15-Octadecatetraenoic acid	Linoleic acids	HMDB0032672	M+H	C18H28O2	-0.2
**M24**	Alpha-Linoleic acid	Linoleic acids	HMDB0001388	M+H	C18H30O2	0.9
**M25**	12 (S)-Leukotriene B4	Eicosanoids	HMDB0005089	M+NH4	C20H32O4	0.0
**M26**	13-cis-Retinoic acid	Lipids	HMDB0006219	M+H	C20H28O2	1.7
**M27**	Inosine	Nucleosides	HMDB0000195	M+H	C10H12N4O5	-2.6

**Figure 5:**
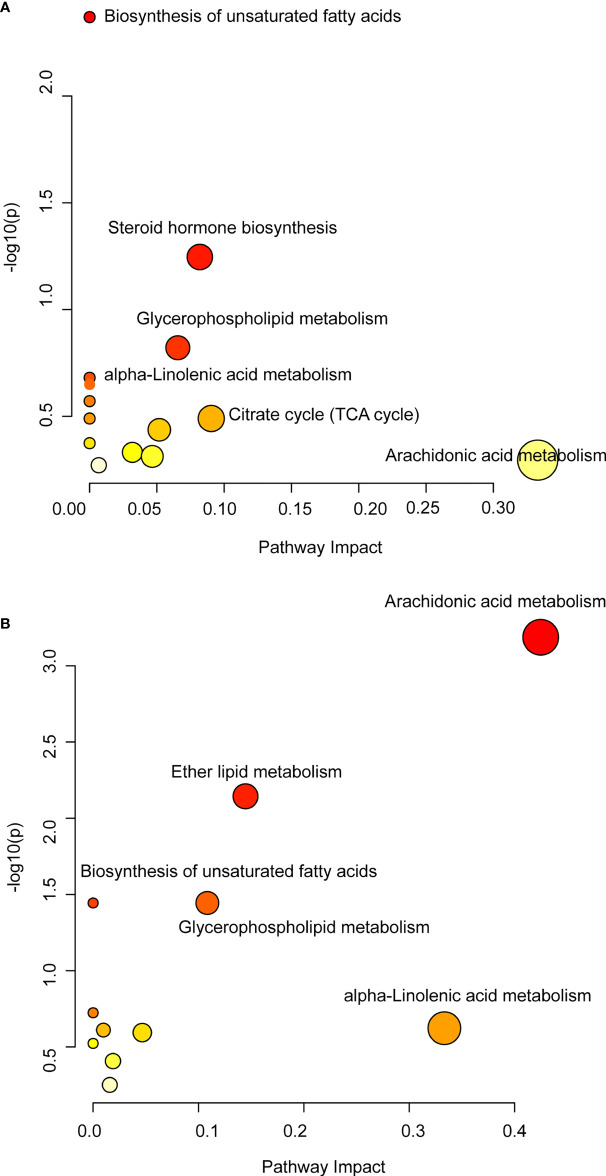
Differential metabolic pathways of DMs. **(A)** Metabolic pathway changes induced by QFPDD (QFPDD vs. Mod) in the lung tissue. **(B)** Metabolic pathway changes induced by QFPDD (QFPDD vs. Mod) in the serum.

In order to further clarify the effect of QFPDD on lipid metabolism-related metabolic pathways, we mapped the pathways based on the DMs obtained from lung tissue and serum. Based on relative abundance of the altered metabolites, the metabolic pathways possibly influenced were searched *via* the KEGG database. Pathway enrichment analysis showed that KEGG metabolic pathways including the biosynthesis of unsaturated fatty acids, steroid hormone biosynthesis, glycerophospholipid metabolism, alpha-linolenic acid metabolism, citrate cycle (TCA cycle), and arachidonic acid metabolism were significantly enriched in the lung tissue after QFPDD treatment (**Figure 5A**). Similarly, QFPDD can significantly improve arachidonic acid metabolism, ether lipid metabolism, biosynthesis of unsaturated fatty acids, glycerophospholipid metabolism, and alpha-linolenic acid metabolism in the serum metabolome (**Figure 5B**). Arachidonic acid metabolism, biosynthesis of unsaturated fatty acids, glycerophospholipid metabolism, and alpha-linolenic acid metabolism were the metabolic pathways significantly associated with immunity and inflammation (Sevastou et al., 2013; Angela et al., 2016; Wang et al., 2019; Wu et al., 2021). The above results suggest that lipid-related metabolic pathways play an important role in the treatment of QFPDD.

### 3.5 Correlation analysis reveals the key feature of *Qing-Fei-Pai-Du* decoction in the microbe–metabolic axis

Based on the obtained microbiome and serum metabolome data, we further conducted Spearman’s correlation analysis to determine the correlation of relevant bacteria and metabolites with immune-inflammation-related indicators after QFPDD treatment.

**Figure 6:**
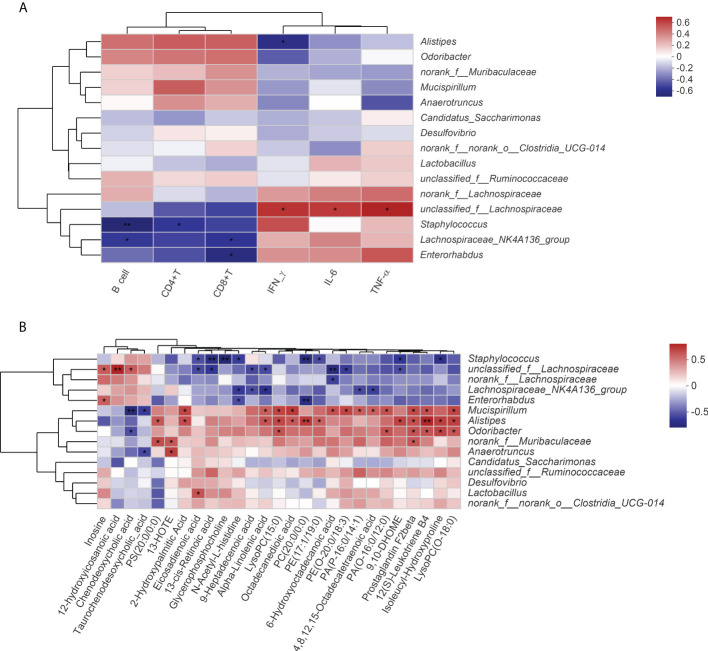
Spearman correlation analysis. **(A)** Correlation analysis of the gut microbiota with immune and inflammatory indicators. **(B)** Correlation analysis of the gut microbiota with serum DMs. Significance levels are indicated as follows: *, p < 0.05; **, p < 0.01.

As shown in **Figure 6A**, multiple immune-related indicators (B cell and CD8^+^ and CD4^+^ T cells) were significantly negatively correlated with multiple gut microbiota (*Staphylococcus*, *Lachnospiraceae_NK4A136_group* and *Enterorhabdus*, *unclassified_f_Lachnospiraceae*), while inflammatory factors (tumor necrosis factor (TNF-α) interferon gamma (IFN-γ), and interleukin 6 (IL-6)) were positively correlated with them. *Alistipes* and *Odoribacter* were positively correlated with increased level of immune-related indicators (B cell and CD8^+^ and CD4^+^ T cells) in the QFPDD group. In addition to being significantly correlated with immune and inflammatory indicators, gut microbiota was also significantly correlated with a variety of metabolites. As shown in **Figure 6B**, *Staphylococcus*, *unclassified_f_Lachnospiraceae*, *Lachnospiraceae_NK4A136_group*, and *Enterorhabdus* were significantly negatively correlated with the metabolites of fatty acids and glycerophospholipids; *Mucispirillum*, *Alistipes*, and *Odoribacter* were positively correlated with the metabolites of fatty acids and glycerophospholipids. The results suggest that the immune-enhancing effect of QFPDD is associated with specifically altered microbe–metabolic axis.

## 4 Discussion

COVID-19 is a respiratory disease caused by SARS-CoV-2, which has developed into a global pandemic with high mortality. The pathophysiological feature of SARS-CoV-2 infection is aggressive inflammatory response, which is closely related to multiple organ dysfunction in some patients (Tay et al., 2020). Recently, our study showed that HcoV-229E combined with cold-damp environment could successfully induce the mouse model of pneumonia, and the characteristics of the animal model were similar to the clinical characteristics of COVID-19 patients (Tian et al., 2022).

Previous studies have shown a correlation between plasma concentrations of several chemokines and cytokines and gut microbiota composition in COVID-19 patients, suggesting that the gut microbiota could play a role in regulating the host immune response and potentially influence disease severity and prognosis (Zuo et al., 2020; Yeoh et al., 2021). The metabolome and lipidome revealed widespread metabolic dysregulation in COVID-19 patients associated with gut microbiota disturbances (Lv et al., 2021; Ren et al., 2021). Due to the complexity of TCM, their therapeutic mechanisms may be more difficult to uncover, and a multi-omics approach opens new doors to expand our understanding of these associations. Clinically, the immune function, pulmonary inflammatory response, and multiorgan damage can be significantly improved after QFPDD treatment (Xin et al., 2020). In our previous study, we also found that QFPDD could improve immune function and reduce liver injury and inflammatory response in pneumonia model mice by liver single-cell RNA sequencing (Tian et al., 2022). However, whether the effect of QFPDD on improving the immune function and reducing inflammation were associated with gut microbiota remains unclear. To further reveal the potential mechanism of the effect of QFPDD treatment on the immune system of COVID-19 patients, we investigated the regulatory effects of QFPDD on gut microbiota and metabolome. Our study showed that QFPDD could improve the gut microbiota and metabolic disorders of pneumonia model mice, thereby improving the immune function and reducing the inflammatory response in the model mice.

Studies have shown that pulmonary infections caused by respiratory viral infections such as influenza and respiratory syncytial virus can alter the gut microbiome (Wang et al., 2014; Yildiz et al., 2018). Viral infections easily cause secondary bacterial infections in patients, which often have a more severe clinical course (Brundage, 2006; Hanada et al., 2018). Recently, studies have provided direct evidence that altered fecal microbiota is associated with fecal SARS-CoV-2 levels and COVID-19 severity, and that the gut microbiota might be associated with COVID-19 severity by modulating host immune responses; strategies to alter the gut microbiota might reduce disease severity (Zuo et al., 2020; Yeoh et al., 2021). In previous studies, Firmicutes were found to be the most significantly associated with COVID-19 disease severity (15 of 23 bacterial taxa), such as *Alistipes*, *Staphylococcus*, and *Lanchnospiraceae* (Zuo et al., 2020); fecal samples with low to no SARS-CoV-2 infectivity had higher abundance of *Alistipes* and *Lanchnospiraceae* compared with those with high SARS-CoV-2 infectivity (Zuo et al., 2021). In our study, we found that Firmicutes were significantly associated with the efficacy of QFPDD and showed a significant correlation with immune and inflammatory-related indicators, especially *Alistipes*, *unclassified_f_Lachnospiraceae*, *Lachnospiraceae_NK4A136_group*, and *Staphylococcus*. *Alistipes* species are indole positive, involved in tryptophan metabolism of serotonin precursor and maintaining gut immune homeostasis, and positively correlated with B-cell precursors (Gao et al., 2018), which was consistent with our study. *Lachnospiraceae* bacteria can produce short-chain fatty acids, which play an important role in enhancing host immunity (Klaring et al., 2015; Ratajczak et al., 2019). In view of the limited therapeutic effect on COVID-19, understanding the host cytokine pathway and the interaction between microbial community and cytokine responses in SARS-CoV-2 infection is essential for the development new therapeutic methods (Mendes et al., 2019). Although we cannot determine the specific mechanism of these bacteria in the pathogenesis of disease and QFPDD treatment, our data highlight the potential role of bacteria in QFPDD treatment of COVID-19 patients.

In addition to gut microbiota, metabolites also play an important role in the progression of COVID-19. Previous studies have shown that patients with COVID-19 have obvious metabolic disorders, including glycerophospholipids, fatty acids, diacylglycerols, and sphingomyelins (Song et al., 2020). Glycerophospholipids were most affected by QFPDD in serum and lung metabolome, which is in line with the metabolomic analysis of patients with COVID-19 recently published in *Cell* (Shen et al., 2020). The affected glycerophospholipids included phosphatidylcholine (PC), lysophosphatidylcholine (LysoPC), phosphatidylethanolamines (PE), lysophosphatidylethanolamine (LysoPE), and lysophosphatidic acid (LysoPA). LysoPC and LysoPA are the most bioactive hemolytic phospholipids and are key signaling molecules in cell and tissue metabolism involved in plasma membrane formation, cell growth and apoptosis, and inflammatory response (Makide et al., 2009; Sevastou et al., 2013; Shindou et al., 2013). They have also emerged as a novel class of inflammatory lipids, joining thromboxane, leukotriene, and prostaglandin, with which they share metabolic pathways and regulatory mechanisms (Sevastou et al., 2013). In addition to glycerophospholipids, fatty acids, which play a key role in the development of inflammation and immunity, were also significantly affected by QFPDD treatment (Calder et al., 2009; Innes and Calder, 2018; Saini and Keum, 2018). In our study, multiple fatty acids were significantly associated with inflammatory (IFN-γ, IL-6, and TNF-α) and immune (CD4^+^T, CD8^+^T, and B cells) indicators, including eicosadienoic acid, alpha-linolenic acid, 12-hydroxyicosanoic acid, and octadecanedioic acid. The results of pathway enrichment analysis also showed that biosynthesis of unsaturated fatty acids, alpha-linolenic acid metabolism, and arachidonic acid metabolism were significantly changed in serum and lung tissue after QFPDD treatment. In a recent study, fatty acid biosynthesis, arachidonic acid metabolism, and alpha-linolenic acid and linoleic acid metabolism were also found to be altered in COVID-19-positive or COVID-19-negative patients compared with healthy controls (Castane et al., 2022). Among the metabolites related to the efficacy of QFPDD, inosine, a nucleotide metabolite, has a significantly positive correlation with inflammatory factors (IL-6 and TNF-α). In the non-severe COVID-19 group, inosine was negatively correlated with platelet, white blood cell (WBC), and monocyte counts (Schultz et al., 2022). The increased levels of inosine could be caused by the body’s attempt to resolve the immune thrombosis acting on immune cells and fighting against the excessive inflammation. These actions of inosine have been previously reported, but the mechanism of action remains unclear (Marton et al., 2001; Fuentes et al., 2014; Sliva et al., 2019).

Finally, the correlation analysis of the gut microbiome, metabolome, and immune-inflammatory indicator axis showed the overall correlativity during QFPDD treatment. The significantly altered gut taxa, metabolites, and disease indicators explain the therapeutic mechanism of QFPDD in COVID-19. QFPDD attenuates symptoms in patients with COVID-19 through overall changes in the body, especially through the crosstalk between gut microbiota and accompanying host metabolites. We first studied the therapeutic mechanism of QFPDD in the treatment of COVID-19 from the perspective of the gut microbiome, metabolome, and their relationship.

## 5 Conclusion

To the best of our knowledge, this is the first report of integrated microbiome and metabolomic study of QFPDD in the treatment of pneumonia model mice. Our study showed that the link between metabolic biomarkers and gut microbiota has an important on the efficacy of QFPDD. QFPDD could improve the immune function and reduce the inflammatory response of pneumonia model mice by remodeling the gut microbiota and its corresponding host co-metabolism. The study provides a new insight into the clinical treatment of COVID-19 with QFPDD from the perspective of gut microbiota-metabolism-phenotype axis. On the other hand, the integrated multi-omics analysis method was demonstrated to be effective for better understanding complex traditional medicine.

## Data availability statement

The datasets presented in this study can be found in online repositories. The names of the repository/repositories and accession number(s) can be found below: BioProject: PRJNA839182.

## Ethics statement

Animal protocols were reviewed and approved by the Laboratory Animals Ethics Committee of Chinese Materia Medica, China Academy of Chinese Medical Sciences (approval code: 2020D028).

## Author contributions

WZ (Weidong Zhang) and HL co-supervised the project. GW, WZ (Wendan Zhang), and NZ analyzed the data and drafted this manuscript. XZ, ST, JZ, YZ, JL, and LS helped analyzing the data, GG provided the chemical profiling results of QFPDD. All authors contributed to the article and approved the submitted version.

## Funding

The work was supported by the National Key Research and Development Program of China (2020YFC0845400), National Natural Science Foundation of China (82141203, 82004003, 82004215), Shanghai Municipal Science and Technology Major Project (ZD2021CY001), Innovation Team and Talents Cultivation Program of National Administration of Traditional Chinese Medicine (ZYYCXTDD-202004), Shanghai Sailing Program (20YF1459000), Shanghai Municipal Health Commission Project (20204Y0326), Shanghai Municipal Science and Technology Major Project (ZD2021CY001).

## Acknowledgments

We are very grateful to Prof. Xiaolan Cui and her team from the Chinese Academy of Chinese Medical Sciences (ABSL-2 Laboratory) for their assistance in establishing the animal model.

## Conflict of interest

The authors declare that the research was conducted in the absence of any commercial or financial relationships that could be construed as a potential conflict of interest.

## Publisher’s note

All claims expressed in this article are solely those of the authors and do not necessarily represent those of their affiliated organizations, or those of the publisher, the editors and the reviewers. Any product that may be evaluated in this article, or claim that may be made by its manufacturer, is not guaranteed or endorsed by the publisher.
