# Supplementary material for: Integrated microbiome and metabolome analysis reveals the potential therapeutic mechanism of Qing-Fei-Pai-Du decoction in mice with coronavirus-induced pneumonia
Source: Front Cell Infect Microbiol. 2022 Aug 26;12:950983. doi: 10.3389/fcimb.2022.950983 (PMC9461713; doi:10.3389/fcimb.2022.950983)
Supplement: Supplementary file 2 [file DataSheet_2.docx]

Supplementary Material

## 1. Detection of HCoV-229E in lung tissue

The lung tissue was placed in a mortar and a small amount of liquid nitrogen was poured into it when the tissue was ground into powder with a pestle. The powder was placed in a 1.5 mL centrifuge tube, 1 mL of TRIzol reagent was added. The powder was repeatedly blown and suspended and incubated for 20 min at room temperature for full lysis, then centrifuged at 4 ℃ for 10 min at 12 000 r·min-1. The supernatant was transferred into another tube and added with 0.2 mL of chloroform and shaken vigorously for 15 s. The samples were kept for standing for 5 min until the liquid was stratified. The samples were centrifuged at 4 ℃ at 12,000 r·min-1 for 15 min. The supernatant was transferred into another tube and added with 0.5 mL of isopropanol, after kept for standing for 30 min at room temperature, the sample was centrifuged at 12,000 r·min-1 for 10 min at 4 ℃.The supernatant was discarded, and the sediment was washed with 1 mL of 75% ethanol and centrifuged at 4 ℃ at 7 500 r·min-1 for 5 min. The supernatant was sucked up, and the residue was dissolved in 20 μL of DEPC water. The expression of HcoV-229E nucleic acid was detected with HCoV-229E Real-time RT-PCR kit according to the protocol.

# 2 Serum and lung tissue sample preparation and metabolome analysis

## 2.1 Materials and chemical

LC-MS grade acetonitrile, methanol, water, ammonium formate and formic acid were purchased from Fisher Scientific (Fair Lawn, NJ, US).

## 2.2 Sample preparation

For serum samples, an aliquot of 50 μL of thawed serum sample was deproteinized with 150 μL of MeOH:CAN (1:1, v/v) precooled to -20 ℃. After vortex for 30 s and sonication for 10 min in an ice bath, samples were overnight at -20 ℃ to improve protein precipitation and then centrifuged at 12, 000 g for 15 min at 4 ℃, 2 μL of supernatant was subjected to HPLC-QTOF-MS/MS analysis. For the lung tissues, 10 mg of tissue was accurately weighed, 200 μL of water was added and homogenized by tissue homogenizer, then 800 μL of ACN: MeOH (1:1, v/v) was added. After vortexed for 30 s, samples were treated with ultrasound for 10 min on an ice bath and then overnight at -20 ℃ refrigerator, and finally centrifuged at 12, 000 g for 15 min at 4 ℃. The supernatant was transferred into a clean dry tube and dry it with nitrogen at 30 ℃, residue was reconstituted with 100 μL of ACN: H2O (1:1, v/v), vortexed for 30 s, sonicated for 5 min in an ice bath, then centrifuged at 12, 000 g for 15 min at 4 ℃. 2 μL of supernatant was subjected to HPLC-QTOF-MS/MS analysis.

## 2.3 HPLC-QTOF-MS/MS analysis

Sample analysis was performed on a Shimadzu HPLC system equipment with SCIEX Triple TOF 5600+. The chromatographic column was ACQUITY UPLC BEH C18 column (2.1 × 100 mm, 1.8 µm). The mobile phase A consists of 0.1% formic acid in H2O, mobile phase B was ACN. The gradient was used as follows: 1% B, 0–1.5 min; 1%~99% B, 1.5–13 min; 99% B, 13–16.5 min; 99%~1% B, 16.5–16.6 min; 1% B, 16.6–20 min. The column temperature was 40 °C, flow rate was 0.3 ml/min, and the volume of injection was 2 µl for each run.

The metabolomics profiling analysis was performed on an SCIEX Triple TOF 5600+ with information dependent acquisition (IDA). For the positive mode, the collision energy (CE) spread was set as 40 eV and 10 eV, declustering potential (DP) set at 60 V, the ion spray voltage floating (ISVF) set at 5500 V, and the temperature set to 550ºC. For the negative mode, the collision energy (CE) spread were set as -40 eV and -10 eV, declustering potential (DP) set at -60 V, the ion spray voltage floating (ISVF) set at 4,500 V, and the temperature set at 450ºC. The other source same parameters settings in the two modes were as follows: the ion source gas1 and gas2 were set at 60 psi with curtain gas was set at 35 psi, the TOF/MS full scan was operated with the mass range was 60-1000 Da and the TOF-MS/MS full scan was operated with the mass range was 25-1000 Da, and the accumulation time was 0.15 s. The mass spectrometer was automatically calibrated by the calibration delivery system (CDS) once every 13 injections.

## 2.4 Data processing and analysis

The raw data were imported to the Progenesis QI for peak alignment to obtain the peak area list and the identification result list. Then statTarget was used to signal drift correction and normalize the data. The CV (coefficient of variation) of metabolites in the QC samples was set at a threshold of 30%, as a standard in the assessment of repeatability in metabolomics data sets. The nonparametric univariate method (Mann-Whitney-Wilcoxon test) was used to analysis metabolites that differed in abundance between the different subgroups to ensure that the peak of each metabolite was reproducibly detected in the samples. Then, the peak list was imported into SIMCA 14.0 software (Umetrics AB, Umea, Sweden) to acquire clustering information and important variable. The online HDMB data (https://hmdb.ca/), LIPIDMAPS (https://www.lipidmaps.org/), and METLIN (https://metlin.scripps.edu) were used to align the molecular mass data to identify metabolites. the mass error used was 10 ppm for MS1 and 5 ppm for MS2. Metabolites annotated were subjected to further statistical analysis by using MetaboAnalyst 5.0 (https://www.metaboanalyst.ca/), the differential metabolites were used to conduct hierarchical cluster analyses (HCA) and mapped into their biochemical pathways, pathway information was obtained from the KEGG.

# 3 16S rRNA sequencing

## 3.1 DNA extraction and PCR amplification

Microbial community genomic DNA was extracted from 15 samples using the E.Z.N.A.® soil DNA Kit (Omega Bio-tek, Norcross, GA, U.S.) according to manufacturer’s instructions. The DNA extract was checked on 1% agarose gel, and DNA concentration and purity were determined with NanoDrop 2000 UV-vis spectrophotometer (Thermo Scientific, Wilmington, USA). The hypervariable region V3-V4 of the bacterial 16S rRNA gene were amplified with primer pairs 338F (5'-ACTCCTACGGGAGGCAGCAG-3') and 806R(5'-GGACTACHVGGGTWTCTAAT-3') by an ABI GeneAmp® 9700 PCR thermocycler (ABI, CA, USA). The PCR amplification of 16S rRNA gene was performed as follows: initial denaturation at 95 ℃ for 3 min, followed by 27 cycles of denaturing at 95 ℃ for 30 s, annealing at 55 ℃ for 30 s and extension at 72 ℃for 45 s, and single extension at 72 ℃ for 10 min, and end at 4 ℃. The PCR mixtures contain 5 × TransStart FastPfu buffer 4 μL, 2.5 mM dNTPs 2 μL , forward primer (5 μM) 0.8 μL, reverse primer (5 μM) 0.8 μL, TransStart FastPfu DNA Polymerase 0.4 μL, template DNA 10 ng, and finally ddH2O up to 20 μL. PCR reactions were performed in triplicate. The PCR product was extracted from 2% agarose gel and purified using the AxyPrep DNA Gel Extraction Kit (Axygen Biosciences, Union City, CA, USA) according to manufacturer’s instructions and quantified using Quantus™ Fluorometer (Promega, USA).

## 3.2 Illumina MiSeq sequencing

Purified amplicons were pooled in equimolar and paired-end sequenced on an Illumina MiSeq PE300 platform/NovaSeq PE250 platform (Illumina, San Diego,USA) according to the standard protocols by Majorbio Bio-Pharm Technology Co. Ltd. (Shanghai, China). The raw reads were deposited into the NCBI Sequence Read Archive (SRA) database (Accession Number: PRJNA839182).

## 3.3 Processing of sequencing data

The raw 16S rRNA gene sequencing reads were demultiplexed, quality-filtered by fastp version 0.20.0 and merged by FLASH version 1.2.7 with the following criteria: (i) the 300 bp reads were truncated at any site receiving an average quality score of <20 over a 50 bp sliding window, and the truncated reads shorter than 50 bp were discarded, reads containing ambiguous characters were also discarded; (ii) only overlapping sequences longer than 10 bp were assembled according to their overlapped sequence. The maximum mismatch ratio of overlap region is 0.2. Reads that could not be assembled were discarded; (iii) Samples were distinguished according to the barcode and primers, and the sequence direction was adjusted, exact barcode matching, 2 nucleotide mismatch in primer matching.

Operational taxonomic units (OTUs) with 97% similarity cutoff were clustered using UPARSE version 7.1, and chimeric sequences were identified and removed. The taxonomy of each OTU representative sequence was analyzed by RDP Classifier version 2.2[5] against the 16S rRNA database (eg. Silva v138) using confidence threshold of 0.7.

# Supplementary Figures


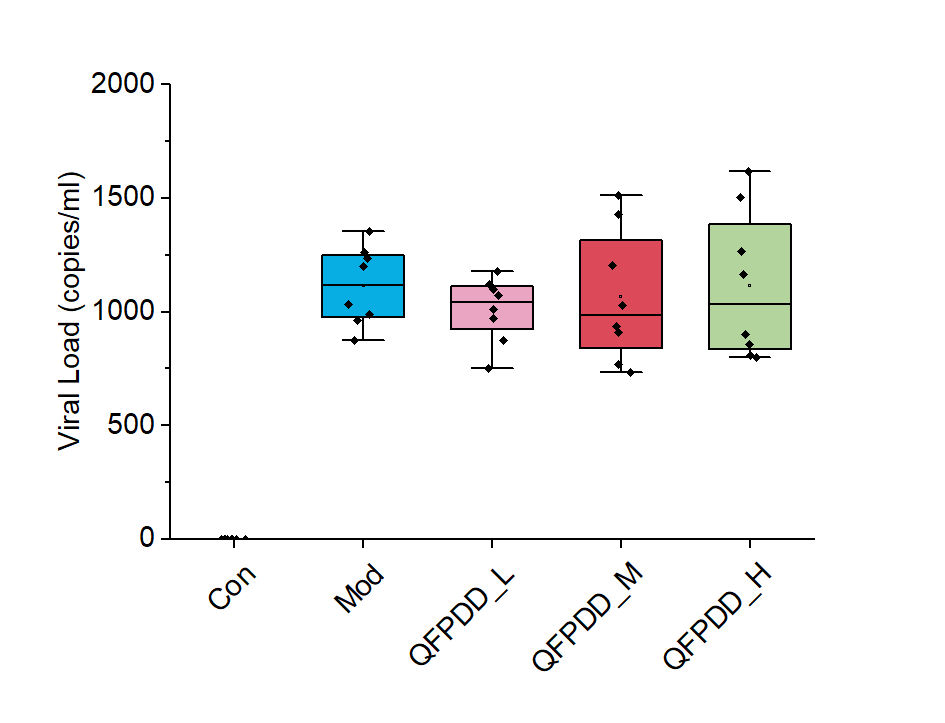


**Figure S1** HCoV-229E viral load


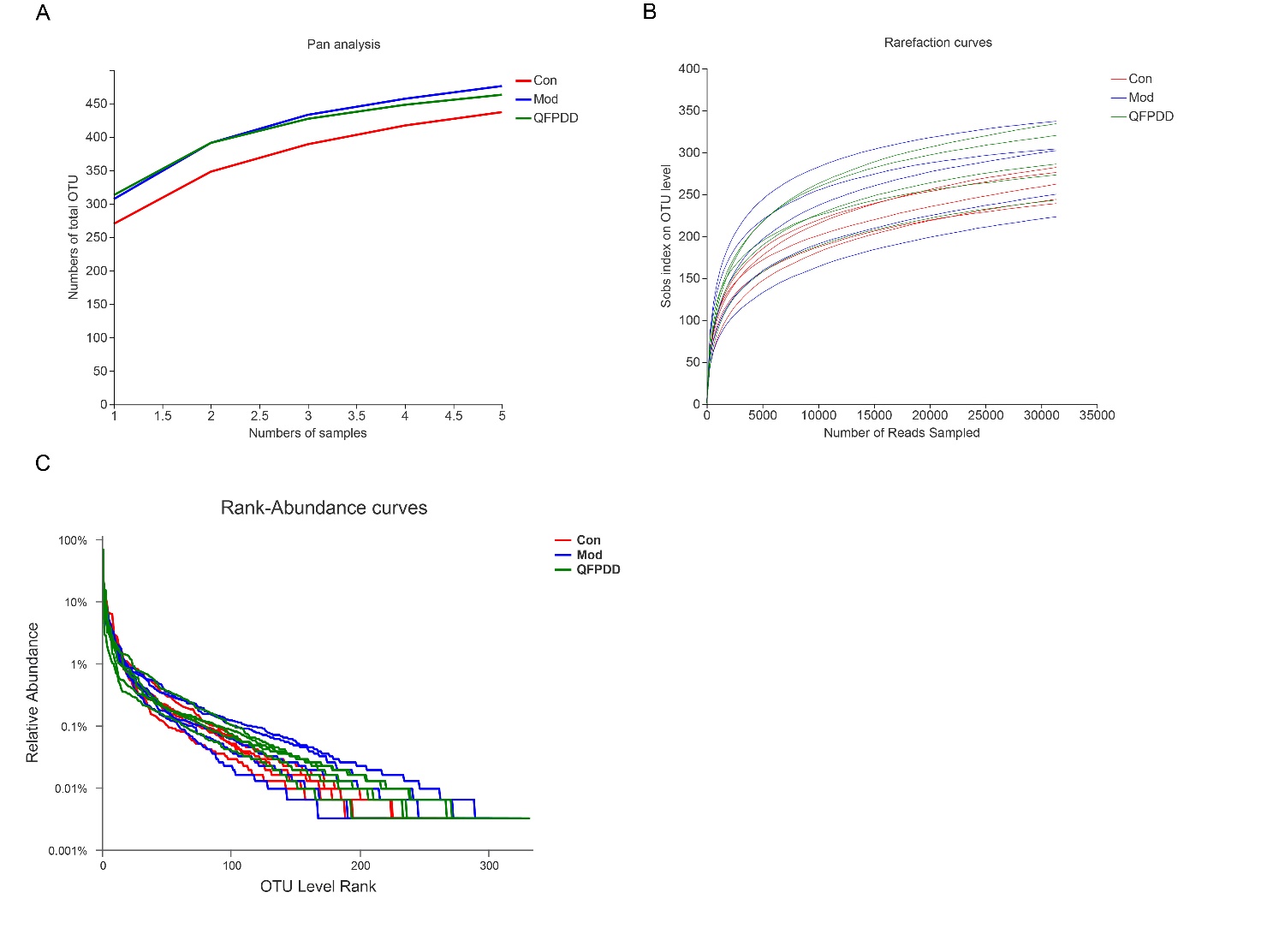


**Figure S2**. Relative bacterial richness and evenness analyses. (A) The species accumulation curve; (B) The rarefaction curve; (C) The rank abundance distribution curves. *n* = 5 for each groups.


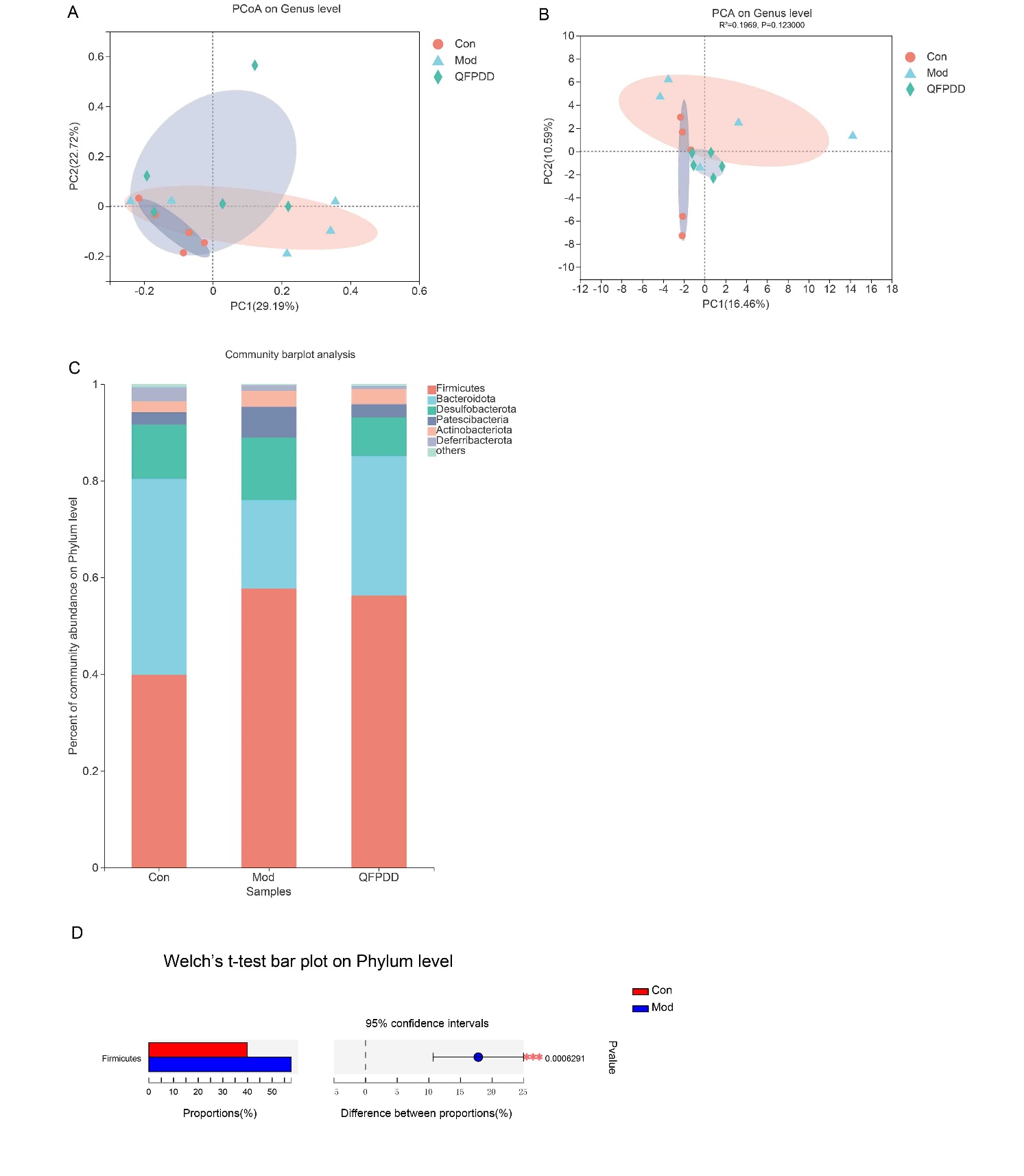


**Figure S3.** Gut microbiome diversity and structure analysis. (A) PCoA plot base of the relative abundance of genus level showing bacterial structural clustering. (B) PCA plot base of the relative abundance of genus level showing bacterial structural clustering. (C) component proportion of bacterial phylum in each group. (D) Welch’s t-test bar plot on Phylum level between Con and Mod groups. (*n* = 5 for each group).


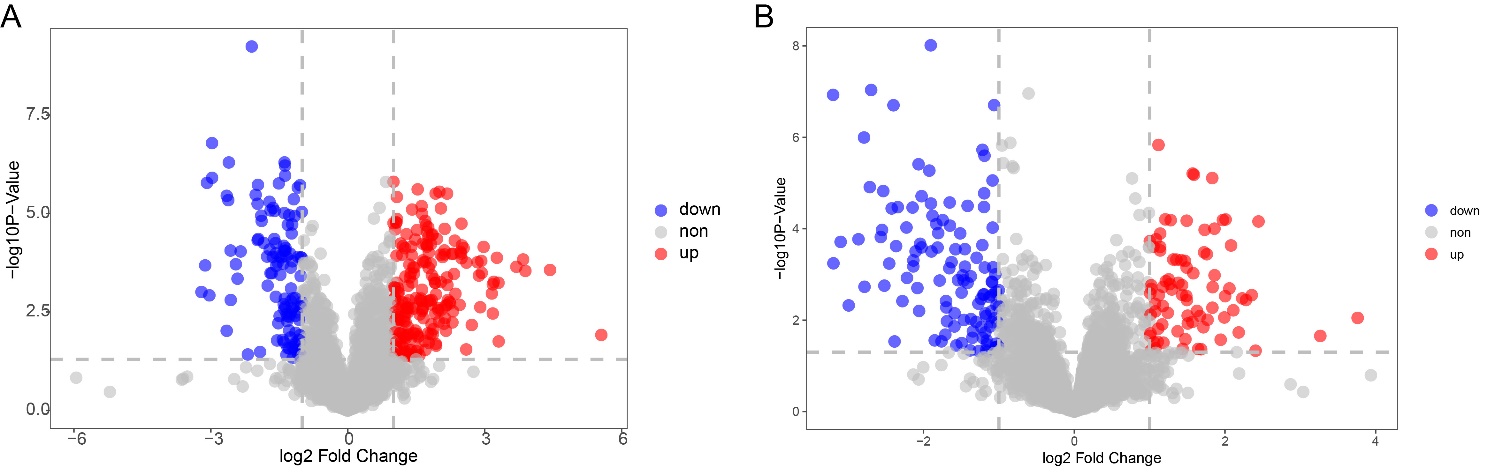


**Figure S4**. Volcano plot showing the differentially accumulated and significantly changed metabolites in the Con and Mod groups. (A) Lung tissue metabolome. (B) serum metabolome.
